# Contents of antenatal care services in Afghanistan: findings from the national health survey 2018

**DOI:** 10.1186/s12889-023-17411-y

**Published:** 2023-12-11

**Authors:** Muhammad Haroon Stanikzai, Essa Tawfiq, Massoma Jafari, Abdul Wahed Wasiq, Mohammad Khaled Seddiq, Sheena Currie, Hadia Sayam, Ahmad Haroon Baray, Sayed Ataullah Saeedzai

**Affiliations:** 1 Department of Public Health, Faculty of Medicine, Kandahar University, District # 10, Kandahar, 3801 Afghanistan; 2 The Kirby Institute, UNSW Sydney, Sydney, Australia; 3 McMaster University, Hamilton, Ontario Canada; 4 Department of Internal Medicine, Faculty of Medicine, Kandahar University, Kandahar, Afghanistan; 5 Stop Tuberculosis Partnership of Afghanistan, Kabul, Afghanistan; 6 Jhpiego, 1615 Thames Street, Baltimore, MD 21231 USA; 7 Department of Para-clinic, Faculty of Medicine, Malalay Institute of Higher Education, Kandahar, Afghanistan; 8 Ministry of Public Health, Kabul, Afghanistan

**Keywords:** Afghanistan, Antenatal care, ANC contents, Pregnant women, AHS

## Abstract

**Background:**

Maternal and newborn mortality is a public health concern in low- and middle-income countries (LMICs), including Afghanistan, where the evolving socio-political circumstances have added new complexities to healthcare service delivery. Birth outcomes for both pregnant women and their newborns are improved if women receive benefits of quality antenatal care (ANC).

**Objectives:**

This study aimed to assess the contents of ANC services and identify predictors of utilization of services by pregnant women during ANC visits to health facilities in Afghanistan.

**Methods:**

In this cross-sectional study, we used data from the Afghanistan Health Survey 2018 (AHS2018). We included a total of 6,627 ever-married women, aged 14–49 years, who had given birth in the past 2 years or were pregnant at the time of survey and had consulted a health worker for ANC services in a health facility. The outcome was defined as 1–4 services and 5–8 services that a pregnant woman received during an ANC visit. The services were (i) taking a pregnant woman’s blood pressure, (ii) weighing her, (iii) testing her blood, (iv) testing her urine, (v) providing advice on nutrition, (vi) advising about complicated pregnancy, (vii) advising about the availability of health services, and (viii) giving her at least one dose of Tetanus Toxoid (TT) vaccine. The binary outcome (1–4 services versus 5–8 services) was used in a multivariable logistic regression model.

**Results:**

Of all 6,627 women, 31.4% (2,083) received 5–8 services during ANC visits. Only 1.3% (86) received all 8 services, with 98.7% (6,541) receiving between 1 and 7 services, and 71.6% (4,745) women had their blood pressure measured during ANC visits. The likelihood (adOR = Adjusted Odds Ratio) of receiving 5–8 services was higher in women who could read and write (adOR = 1.33: 1.15–1.54), in women whose husbands could read and write (adOR = 1.14: 1.00-1.28), in primipara women (adOR = 1.42: 1.02–1.98), in women who knew one danger sign (adOR = 5.38: 4.50–6.45), those who knew 2 danger signs (adOR = 8.51: 7.12–10.19) and those who knew ≥ 3 danger signs (adOR = 13.19: 10.67–16.29) of complicated pregnancy, and in women who had almost daily access to TV (adOR = 1.16: 1.01–1.33). However, the likelihood of receiving 5–8 services was lower in women who used private clinics (adOR = 0.64: 0.55–0.74) and who received services from nurses (adOR = 0.27 (0.08–0.88).

**Conclusion:**

Our findings have the potential to influence the design and implementation of ANC services of health interventions to improve the delivery of services to pregnant women during ANC visits.

**Supplementary Information:**

The online version contains supplementary material available at 10.1186/s12889-023-17411-y.

## Introduction

Antenatal care (ANC) offers a unique and vital opportunity to “prevent, detect, and treat” complications that may arise during pregnancy [1]. The WHO recognizes the crucial role of ANC in maternal and neonatal health as well as in reducing stillbirths, and underscores the necessity for a minimum of eight visits during pregnancy [2–4]. These visits can commence preferably before or as early as the 12th week of gestation, offering timely interventions that ensure optimal health outcomes [2–4]. While the benefits of ANC are widely acknowledged, disparities exist in its accessibility, service content, quality, and uptake across different regions, particularly in countries grappling with socio-economic and political challenges, adding another layer of complexity to its healthcare challenges [3, 5–7].

One of the main concerns is the delivery and contents of ANC services in low- and middle-income countries (LMICs). Without suitable contents, these connections largely remain “missed opportunities” [8]. A comprehensive survey across 10 LMICs showed that the total of recommended ANC components was availed by a mere 10–50% of participating women [9]. Extending this narrative, a study encompassing 91 developing nations ascertained that approximately 53.8% of pregnant women accessed only three of the recommended components of ANC services [10]. Such variances in providing ANC services have profound implications for both maternal and neonatal health outcomes [3, 11, 12].

The components and contents of ANC are intrinsically linked to its overall quality, with comprehensive and evidence-based contents contributing to superior care [13]. Four ANC visits may be insufficient to ensure that the recommended ANC interventions are implemented [14].

Research indicates that a multitude of factors significantly influence the quality (and subsequently the contents) of ANC services provided to pregnant women. Determinants such as maternal age, employment status, marital status, geographical residency, educational attainment, parity, ethnicity, household economic standing, spouse’s educational level, location of the ANC service point, proximity to a healthcare facility, exposure to media channels, expenses, the attitudes and professionalism of the ANC provider, the timing of ANC, the frequency of ANC consultations, the duration of wait times, and the overall client satisfaction have been identified as pivotal in shaping the comprehensiveness and robustness of ANC services’ quality and contents [14–19].

In a country like Afghanistan, which faces one of the world’s highest maternal mortality rates at 638 per 100,000 live births [20, 21] and an infant mortality rate of 36 per 1,000 live births [22], optimizing ANC services is vital. In spite of three decades of dedicated efforts by both national and international community, Afghanistan faces significant challenges in achieving the recommended eight ANC visits. These challenges stem from its prolonged conflict, persistent political instability, socio-economic disparities, and under-resourced health infrastructure [23]. Data from 2015 reveals that only 59% of pregnant women accessed ANC services at least once during their pregnancy [24, 25]. Despite WHO’s recommendation of eight ANC visits, in 2018 only 21% of Afghan pregnant women accessed four ANC sessions [26–28]. The low uptake of at least four ANC sessions underscores a critical issue; there is little to gain in pushing for an increase to eight visits when the existing structure fails to retain women through even half of the recommended visits. This is one reason why the standards for reproductive health services in Afghanistan and the WHO both recommend at least four ANC visits [21, 28]. Efforts must initially focus on improving retention for the first four visits before expanding to the eight-visit schedule. Access to ANC services in Afghanistan is highly unequal, with a 25-percentile coverage gap between the country’s 20% poorest and 20% richest, highlighting deep-rooted equity issues [27]. This divide not only reflects disparities in service use but also broader socio-economic influences affecting the quality and accessibility of antenatal care.

In addition, research in Afghanistan indicates that several factors significantly influence ANC service utilization. These include the spouse’s educational background, women’s literacy rates, their employment status, financial standing, place of residence, and access to private transportation [21].

Reducing maternal and newborn mortality are global priorities outlined in the Sustainable Development Goals (SDGs) and detailed in the Ending Preventable Maternal Mortality (EPMM) and Every Newborn Action Plan (ENAP) goal, which include reaching 90% coverage of four or more antenatal care visits to improve maternal and newborn health (MNH) outcome [29]. This goal helps focus the pivotal role of ANC recognized as a cost-effective and high-impact intervention in bolstering maternal and newborn health, particularly in resource-limited settings, and cannot be overstated [3]. While literature reports on the adequacy and timing of ANC visits in Afghanistan [26, 30–32], there is a notable gap in in-depth studies focusing on the comprehensive contents of recommended ANC services in this context. To bridge this research void, the present study was designed to examine the contents of ANC services. It also seeks to discover the sociodemographic factors that influence the utilization of ANC services, leveraging secondary data analysis from the Afghanistan Health Survey 2018 (AHS 2018).

The Afghanistan Health Survey conducted in 2018, captured a period when international communities were actively supporting Afghanistan’s reproductive health initiatives. However, at the time of writing this paper, the situation for women has deteriorated significantly. In the current sociopolitical context, women’s access to essential services like education, employment, and travel has been severely restricted [33–35]. Recognizing the pre-existing challenges, it becomes even more critical to identify gaps in ANC services to devise effective interventions and implementation approaches. As an example, poor retention in ANC has necessitated the exploration of group-based ANC models as a potential solution, particularly in LMICs [36, 37].

## Materials and methods

### Data and sample

This was a cross-sectional study for which we used data collected for the AHS 2018 [28]. Details of the survey, including sampling approaches, are provided elsewhere [28]. During the survey, in the selected households, women were interviewed by trained female surveyors who used a questionnaire on the utilization of health services for women. Moreover, women were asked questions as to whether they watched TV, used the internet, and listened to the radio, with response options of (a) almost daily, (b) at least once a week, and (c) never, for each of the three above questions. The head of the household was interviewed on the ownership of household assets (such as TV and radio), construction material used for the property, and household use of amenities for daily life.

In our study, the inclusion criteria were defined as an ever-married woman, aged 14–49 years, who had given birth in the past 2 years (prior to the survey) or was currently pregnant (at the time of survey) and was consulted by a health worker during ANC visits in a health facility. We used data from 6,627 women out of the 6,862 women who had given birth in the past 2 years or were currently pregnant. Out of the 6,862 women, 49 were excluded because they reported that they received ANC services from a health post or community health worker, or traditional birth attendant, and 186 women were excluded because they did not report a specific service related to maternal health during ANC visit(s) from a health facility (see Additional file 1).

The services during ANC visits were defined as (i) taking a pregnant woman’s blood pressure, (ii) weighing her, (iii) testing her blood, (iv) testing her urine, (v) providing her advice on nutrition, (vi) advising her about complicated pregnancy, (vii) advising her about the availability of health services, and (viii) giving her at least one dose of Tetanus Toxoid (TT) vaccine.

### Statistical analysis

#### Outcome variable

The outcome (binary) was defined as 1–4 services versus 5–8 services a pregnant woman received during an ANC visit from a health facility.

#### Predictors

The predictors (explanatory variables) included in the analysis were: woman’s age (14–29 years, 30–39 years, and 40–49 years), woman’s literacy (whether or not she could read and write), husband’s literacy (whether or not he could read and write), parity (the woman had not given birth [nullipara], the woman had given birth once [primipara], the woman had given ≥ 2 births [multipara]), type of health facility the woman attended for ANC visits (private clinic, public primary care facility, public hospital), type of health worker the woman consulted with for services during ANC visits (nurse, doctor, midwife), residential area (urban vs. rural [classified by national statistics office based on administrative criteria]) [28], socioeconomical status (quintile from the lowest to highest), woman’s knowledge of danger signs related to pregnancy or childbirth, and access to the internet (whether or not she used the internet daily), access to the radio (whether or not she listened to radio daily), and access to TV (whether or not she watched TV daily).

The variable on women’s knowledge of pregnancy related danger signs and/ or symptoms had four categories: women with no knowledge of danger signs, women who knew 1 danger sign, women who knew 2 danger signs, and women who knew ≥ 3 danger signs. The variable on socioeconomic status was created by applying the principal component analysis, using the household ownership of assets, household use of amenities of life, and construction material used for the household properties. Employing the “pca” function in Stata version 17, the correlation matrix between the variables of household assets, household use of amenities, and construction material used for the household properties was obtained. The first component, which accounts for the largest possible variance in the data related to household assets, amenities, and construction material for the properties, was retained and used to create a quintile of socioeconomic status.

#### Model specification

A binary logistic regression model was used to provide odds ratios (ORs) on the utilization of 1–4 services versus 5–8 services during ANC visits. Results were provided as unadjusted and adjusted ORs. Because data were collected at the household level, we added a random cluster effect in our model estimates to take the clustering effects of data at the household level into account, and to adjust standard errors for the ORs and 95%CI. We did not apply provincial weights because we were not estimating ORs and 95%CI for each province. Data were analyzed in Stata version 17.

## Results

Table 1 shows that there were 6,627 ever-married women, with 68.6% (4,544) in the 1–4 services and 31.4% (2,083) women in the 5–8 services group. Compared with women in the 1–4 services group, the women in the 5–8 services group were more literate, the women’s husbands were more literate, more women were primipara, women had higher knowledge of danger signs during pregnancy, had more access to the media, lived in a rural area, and used more services from midwives and MoPH clinics and hospitals. The above differences were statistically significant. The differences between the two groups in terms of age and socioeconomic status (SES) were not statistically significant; however, of 6,627 women, 62.9% (4,170) were 14–29 years, and 24.9% (1,649) were at the highest versus 13.6% (899) in the lowest SES quintile.

**Fig. 1:**
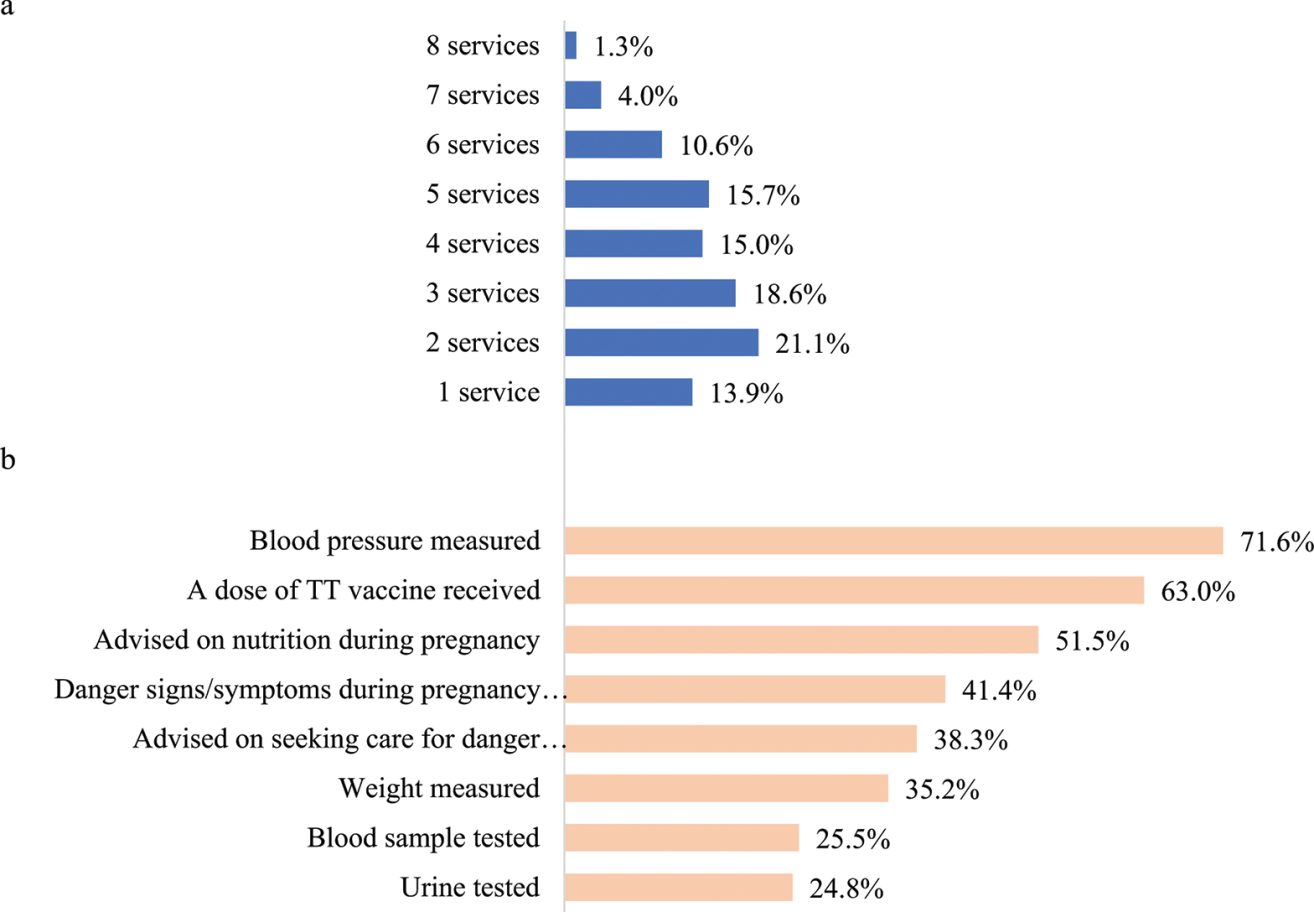
Number and type of ANC services pregnant women received during a single ANC visit. **a** Number of ANC services pregnant women recived during a single ANC. **b** Type of ANC services pregnant women received during a single ANC visit

Figure 1a and Fig. 1b present the number and type of ANC services received. Only 1.3% (86) of pregnant women received all 8 services; 4.0% (265) received 7 services during a single ANC visit, and 94.7% (6,276) received between 1 and 6 services, ranging from 10.6% for 6 services to 21.1% for 2 services. In terms of type of services received in a single ANC visit, 71.6% (4,745) had blood pressure checked; 63.0% (4,175) received one dose of tetanus toxoid vaccine; 51.5% (3,413) received nutritional advice; 41.4% (2,744) were counselled on danger signs; 38.3% (2,538) were advised on seeking care in event of danger signs; 35.2% (2,332) were weighed; 25.5% (1,690) had a blood sample taken; and 24.8% (1,644) had urine taken for testing.

Table 2 presents unadjusted and adjusted odds ratios [unOR, adOR (95%CI)] related to receiving services by pregnant women during their ANC visits to health facilities. It shows that the odds of receiving 5–8 services by women who could read and write was significantly higher compared to women who could not (adOR = 1.33: 1.15–1.54). The likelihood of receiving 5–8 services was higher among women whose husbands could read and write compared to women whose husbands could not (adOR = 1.14: 1.00-1.28). In terms of parity, women who were primipara were more likely to receive 5–8 services compared to nulliparous (adOR = 1.42: 1.02–1.98). The odds of receiving 5–8 services from nurses were significantly lower than receiving services from doctors (adOR = 0.27: 0.08–0.88). It was less likely for pregnant women to receive 5–8 services from private clinics compared to public clinics (adOR = 0.64: 0.55–0.74). In terms of women’s knowledge of danger signs during pregnancy, women who knew the danger sign(s) were more likely to receive 5–8 services compared to women who did not know any danger sign (adOR = 5.38: 4.50–6.45) for those who knew one danger sign; (adOR = 8.51: 7.12–10.19) for those who knew 2 danger signs; and (adOR = 13.19: 10.67–16.29) for women who knew ≥ 3 danger signs. The likelihood of receiving 5–8 services was higher among women with daily access to TV compared to those with no daily access to TV (adOR = 1.16: 1.01–1.33).

**Table 1:** Baseline characteristics of pregnant women who received ANC services

Variables	1–4 services 4,544 (68.6%)	5–8 services 2,083 (31.4%)	Total (%) 6,627 (100%)
Age			
14–29 years 30–39 years 40–49 years	2861 (63.0%) 1375 (30.3%) 308 (6.8%)	1309 (62.8%) 642 (30.8%) 132 (6.3%)	4170 (62.9%) 2017 (30.5%) 440 (6.6%)
**Woman reads and writes	861 (18.9%)	578 (27.8%)	1439 (21.7%)
**Husband reads and writes	1758 (38.7%)	948 (45.5%)	2706 (40.8%)
*Parity			
Nullipara Primipara Multipara	200 (4.4%) 565 (12.4%) 3779 (83.2%)	71 (3.4%) 293 (14.1%) 1719 (82.5%)	271 (4.1%) 858 (13.0%) 5498 (83.0%)
**Health provider			
Nurse Midwife Doctor	24 (0.5%) 2727 (60.0%) 1793 (39.5%)	3 (0.1%) 1497 (71.0%) 601 (28.9%)	27 (0.4%) 4206 (63.5%) 2394 (36.1%)
**Health facility			
MoPH clinic MoPH hospital Private clinic	1640 (36.1%) 662 (14.6%) 2242 (49.3%)	910 (43.7%) 430 (20.6%) 743 (35.7%)	2550 (38.5%) 1092 (16.5%) 2985 (45.0%)
**Number of the danger signs woman knew			
None 1 2 ≥ 3	2066 (45.5%) 1190 (26.2%) 965 (21.2%) 323 (7.1%)	197 (9.5%) 640 (30.7%) 823 (39.5%) 423 (20.3%)	2263 (34.2%) 1830 (27.6%) 1788 (27.0%) 746 (11.3%)
Socioeconomic status			
Lowest (quntile) Low Middle High Highest (quntile)	621 (13.7%) 830 (18.3%) 934 (20.6%) 1031 (22.7%) 1128 (24.8%)	278 (13.4%) 338 (16.2%) 439 (21.1%) 507 (24.3%) 521 (25.0%)	899 (13.6%) 1168 (17.6%) 1373 (20.7%) 1538 (23.2%) 1649 (24.9%)
*Residence			
Urban Rural	1191 (26.2%) 3353 (73.8%)	524 (25.2%) 1559 (74.8%)	1715 (25.9%) 4912 (74.1%)
**Internet			
Almost daily access	133 (2.9%)	93 (4.5%)	226 (3.4%)
*Radio			
Almost daily access	1187 (26.1%)	594 (28.5%)	1781 (26.9%)
**TV			
Almost daily access	1697 (37.4%)	921 (44.2%)	2618 (39.5%)

*p-value < 0.05, **p-value < 0.01

**Table 2:** Likelihood of receiving 5–8 ANC services by pregnant women during a single ANC visit (n = 6,627)

Independent variables	Categories	unOR (95% CI)	P-Value	adOR (95% CI)	P-Value
Age	14–29 years	Ref		Ref	
	30–39 years	1.02 (0.91–1.14)	0.73	1.05 (0.92–1.19)	0.48
	40–49 years	0.94 (0.76–1.16)	0.55	0.90 (0.71–1.13)	0.36
Woman reads and writes	No	Ref		Ref	
	Yes	1.64 (1.45–1.86)	< 0.001	1.33 (1.15–1.54)	< 0.001
Husband reads and writes	No	Ref		Ref	
	Yes	1.32 (1.19–1.47)	< 0.001	1.14 (1.00-1.28)	0.04
Parity	Nullipara	Ref		Ref	
	Primipara	1.46 (1.08–1.98)	0.02	1.42 (1.02–1.98)	0.04
	Primipara	1.28 (0.97–1.69)	0.08	1.13 (0.83–1.53)	0.43
Health provider	Doctor	Ref		Ref	
	Midwife	1.62 (1.44–1.81)	< 0.001	1.09 (0.96–1.25)	0.18
	Nurse	0.37 (0.11–1.24)	0.11	0.27 (0.08–0.88)	0.03
Health facility	MoPH clinic	Ref		Ref	
	MoPH hospital	1.17 (1.01–1.36)	0.04	1.17 (0.99–1.38)	0.06
	Private clinic	0.60 (0.53–0.67)	< 0.001	0.64 (0.55–0.74)	< 0.001
Number of the danger signs the woman knew	None	Ref		Ref	
	1	5.64 (4.73–6.73)	< 0.001	5.38 (4.50–6.45)	< 0.001
	2	8.94 (7.50-10.66)	< 0.001	8.51 (7.12–10.19)	< 0.001
	≥ 3	13.73 (11.17–16.89)	< 0.001	13.19 (10.67–16.29)	< 0.001
Socioeconomic status	Lowest (quntile)	Ref		Ref	
	Low	0.91 (0.75–1.10)	0.33	0.96 (0.78–1.18)	0.68
	Middle	1.05 (0.87–1.26)	0.60	1.13 (0.92–1.38)	0.25
	High	1.10 (0.92–1.31)	0.30	1.08 (0.88–1.32)	0.48
	Highest (quntile)	1.03 (0.86–1.23)	0.73	0.94 (0.73–1.20)	0.61
Residence	Urban	Ref		Ref	
	Rural	1.06 (0.94–1.19)	0.38	1.18 (0.99–1.41)	0.06
Internet	No access	Ref		Ref	
	Almost daily access	1.55 (1.18–2.04)	< 0.001	1.26 (0.92–1.71)	0.15
Radio	No access	Ref		Ref	
	Almost daily access	1.13 (1.00-1.27)	0.04	1.06 (0.93–1.21)	0.35
TV	No access	Ref		Ref	
	Almost daily access	1.33 (1.19–1.48)	< 0.001	1.16 (1.01–1.33)	0.03

Abbreviations: UnOR, Unadjusted Odds Ratio; AdOR, Adjusted Odds Ratio

## Discussion

The importance of assessing the contents of ANC services as a measure of the quality of maternal and newborn health care has been emphasized [13, 14, 19]. Our findings showed that overall, one-third of pregnant women received 5–8 services of the recommended 8 services involved in antenatal care in this nationally representative sample. About 1.3% of pregnant women received all 8 services. Findings for individual contents of antenatal care services received by pregnant women mirrored those reported in studies from other LMICs [13, 16, 17, 19]. These deficits in the contents of antenatal care services have important implications for the health of Afghan pregnant women. It should be noted that in line with changing evidence, clinical recommendations evolve. So the 8 ANC services selected for analysis in the AHS 2018 may no longer be considered as the most important. For example, urine testing may be tailored based on clinical findings and only included if concerns for hypertension and diabetes in pregnancy. Furthermore, the prioritization of certain ANC services may vary depending on the trimester of pregnancy. However, our study was not designed to address the prioritization of these services, or the clinical decision-making related to urine testing.

Although still low, at 31.4%, this proportion of pregnant women who received 5–8 ANC services represents a significant increase from the 16.2% of pregnant women who received 4 ANC services according to the Demographic and Health Survey 2015 (DHS2015) [24, 25]. Previous studies in LMICs used different cut-off points to document the number of recommended ANC services received by a pregnant woman in a single ANC visit [4, 13, 18]. The proportion of women receiving all recommended ANC services ranged from 6.7 to 50% in LMICs [4, 10, 13, 16, 18]. However, in our study, 1.3% of women received all 8 ANC services in a single ANC visit, which may indicate that comprehensive care (where all essential services are provided) ranks among the lowest in Afghanistan as compared with other LMICs. Therefore, our findings illustrate a typical example of a LMIC country with limited resources that lags behind contemporary maternal health frameworks. Recognizing the evolution from the Safe Motherhood Initiative to the current focus on SDGs, the EPMM initiative, and the ENAP, there is a clear imperative to adopt a rights-based, woman-centred approach to care. These progressive frameworks prioritize not only the safety but also the positive experiences of maternal and neonatal care, demanding a more comprehensive, respectful, and rights-based provision of health services for all women [27, 38]. To make meaningful progress towards SDGSs, improving ANC services must be a high priority agenda for the government, UN agencies and health sector stakeholders.

In terms of contents, blood pressure measurement had the highest coverage, with 71.6% among the 8 ANC services. There has been a notable and substantial increase compared to the DHS2015, which showed that 48.8% of pregnant women had blood measured [24, 25]. Our findings are consistent with those from the latest studies in LMICs [9, 17, 19]. For instance, in a recent study, which analyzed data from DHS from 10 LMICs, it was found that blood pressure measurement had the highest coverage across all 10 countries [9]. Likewise, the 2016 Afghanistan National MNH Quality of Care Assessment survey documented that 86% of the patients had their blood pressure checked at an ANC visit [39]. In 2010, the Afghan Mortality Survey showed that pregnancy-related hypertension was the 2nd leading cause of maternal mortality [40]. Consistent and accurate blood pressure monitoring not only serves as a fundamental health check but also stands as a “high impact intervention” and “quality of care indicator” of ANC [38].

Administration of a single dose of tetanus toxoid vaccination is essential at ANC visits; however, this was not the case in 37% of pregnant women in our study. This finding corroborated an earlier report that the tetanus toxoid vaccine was available at 65% of the public institutions in Afghanistan [39]. Tetanus in pregnancy is a major public health problem causing maternal and child deaths in developing countries [41–44]. Hence, targeted interventions ensuring the availability of immunization supplies and competent human resource are needed.

Anemia and pre-eclampsia /eclampsia are major causes of maternal mortality and contributory factors in stillbirth and newborn mortality including Afghanistan [45, 46]. The significance of these conditions is underscored by the fact that a staggering 43% of women aged 15–49 are affected by anemia [27]. However, our study found that the two routine laboratory services to assist in diagnosing these conditions were provided to nearly one quarter of pregnant women during ANC visits; with 24.8% for urine tests and 25.5% for blood tests, which are comparable to the findings of previous studies in LMICs [9, 13, 18]. Additionally, it’s worth noting the alarmingly low provision of iron and folic acid supplements—essential in the prevention of anemia and fetal development issues—where only 7% of pregnant women are receiving these vital nutrients [27]. These findings are concerning and may highlight potential barriers to access and availability of supplies, logistical challenges, funding shortages, shortage of health workers, or other systemic issues that may prevent health facilities from carrying out such tests in LMICs [9, 11, 13]. Equipping health facilities with the capacity to increase the coverage of ANC blood and urine tests is of paramount importance as part of health system strengthening.

Our findings showed that the likelihood of receiving 5–8 ANC services was significantly greater for pregnant women who could read and write. This finding may not be unexpected as previous studies in LMICs demonstrated the importance of women’s education for the increased utilization of health services during ANC visits [47–49]. The ability to read and write is often associated with higher levels of exposure to reading educational materials, watching television, and listening to the radio, which can facilitate understanding of health information and enabling pregnant women to make informed decisions about their health. Therefore, this finding highlights the critical need for Afghan girls and women to not only attend educational facilities but to be fully empowered in accessing health facilities during their pregnancies and discuss with health workers about their health needs for higher quality maternal health services. Recent reports indicate that Afghan women are deprived of their basic human rights, including access to education [35]. This deprivation not only limits their personal growth but also directly impacts their health [33]. Hence, the ban on women’s education should be reversed immediately, and the international community must defend Afghan women’s and girls’ rights to access education.

In this study, women whose husbands could read and write were more likely to receive 5–8 ANC services. This finding was in line with the results of a systematic review undertaken in developing countries [50]. Other studies in Afghanistan have also highlighted the important role of husbands’ education in decision-making regarding seeking healthcare during pregnancy [21, 51]. Consistent with previous studies, a husband with better education may have better understanding of the benefits of ANC services his wife would receive during ANC visits and such husbands are more likely to discuss with their spouses about issues related to women’s reproductive health and maternal health services [13, 21, 50]. These findings urge for a deeper look at gender roles and power dynamics in Afghanistan’s efforts to improve reproductive healthcare. It emphasizes the need for not just women-specific educational programs, but also community-wide interventions that address restrictive cultural norms and promote supportive relationships. Promoting literacy and education among both women and their husbands might be a powerful approach for improving the content of ANC services in Afghanistan, breaking down existing obstacles and contributing to healthier pregnancies and improved MNH outcomes [6, 50].

Consistent with the literature on maternal health, our findings showed that a woman’s parity influences the contents of ANC services she gets from a health provider or facility [4, 52]. In our study, primipara women had higher odds of receiving 5–8 ANC services compared to nullipara pregnant women. A possible reason for this could be that women who have previously given birth have a better understanding of the importance of ANC services and may be more proactive in seeking more ANC services during their contacts with health providers. However, cultural norms in Afghanistan, such as the expectations placed on new brides or first-time mothers, may also impact these associations. The status of a woman in her family and community, control over decision-making, traditional beliefs (about childbirth), and employment can also shape her access to and utilization of MNH services including ANC [21]. Although this finding is critical in understanding the factors influencing the utilization of ANC contents, some other studies have reported that higher parity decreases the likelihood of receiving higher ANC services by pregnant women [53, 54]. More efforts such as awareness and sensitization to nullipara pregnant women are needed to ensure the uptake of more ANC services.

This study found that more than two-thirds of pregnant women (65.8%) knew the danger signs related to pregnancy and labor. Moreover, pregnant women who knew the danger signs of pregnancy were more likely to receive 5–8 ANC services. This finding was in line with a study conducted in Rwanda [55]. Given this finding, it is imperative to disseminate knowledge on the danger signs of pregnancy through public campaigns and health education programs. Incorporating group ANC could be particularly beneficial, as it fosters peer support and learning, where women can share experiences and knowledge about pregnancy complications [37, 56]. Such an approach not only aids in the dissemination of crucial health information but may also enhance the provision and utilization of higher quality ANC services.

Pregnant women consulted by a midwife were more likely to receive 5–8 ANC services than those consulted by a nurse, and this finding is inconsistent with studies from other developing countries [11, 17]. During the prolonged armed conflict and political instability in Afghanistan, most universities were severely impacted, causing an enormous migration of healthcare professionals [57–59]. Women were and still are deprived of their human rights, including professional education and career development, leading to serious shortage of female health professionals per cultural preference [58]. Policymakers responded to the shortage of female health providers by training more healthcare providers through accelerated programs for a rapid expansion of primary care services at the national level [60–62]. Therefore, midwives who received more specialized professional trainings on reproductive health could have led to the delivery of 5–8 ANC services to pregnant women [60, 63]. Since ANC is a fundamental component of a midwife’s role both globally and in Afghanistan, midwives would be afforded the time and opportunity to engage more thoroughly with each pregnant woman [64]. This dedicated time is crucial for conducting comprehensive assessments and having in-depth discussions about the woman’s health needs during ANC visits.

Consistent with relevant literature, our findings indicate that pregnant women who had daily access to TV were more likely to receive 5–8 ANC services than pregnant women who had no access to TV [15, 17]. This is consistent with earlier publications in Rwanda [15], Nigeria [17], and India [11], which showed that access to media was associated with higher chances of utilizing more ANC services. This is perhaps because Afghan national and private broadcasting corporations have been announcing the benefits of receiving maternal healthcare services from health facilities and skilled healthcare providers for years through TV and Radio [26, 65]. These findings highlight the importance of providing pregnant women with access to maternal health messages through various media channels to make informed decisions about their ANC healthcare needs.

Our findings showed a substantial difference in the contents of ANC services provided to pregnant women by private clinics and public health facilities. Pregnant women who received ANC services from a private clinic were less likely to receive 5–8 ANC services in comparison to those who received ANC services from public health facilities. Lack of regulatory bodies to monitor care in the private health sector and low competency of private healthcare providers may explain these associations [66]. Moreover, in the early 2000s, the government of Afghanistan and its allies invested heavily in the construction and renovation of public health facilities for a rapid expansion of primary care services at the national level [61]. Additionally, they implemented several innovative projects aimed at improving the quality of care at the health facilities. It is worth mentioning that the Government needs to be actively involved in monitoring and improving the quality of ANC services in the private health sector as nearly half of the pregnant women (45%) received ANC services from private clinics, according to our findings. The disparities in contents of ANC services underscore the need for robust and effective regulatory mechanisms and quality assurance to ensure safe client care across both public and private healthcare providers [66, 67]. Strengthening regulatory oversight, implementing standards, and enhancing professional education, especially within the private health sector, are crucial steps towards addressing these challenges [58, 66, 68].

While our analysis did not concentrate on a detailed review of ANC service distribution among different wealth tiers, Countdown 2030 report highlights a significant coverage gap in 4 ANC visits between the poorest and wealthiest in society, emphasizing the need for focused research into the underlying reasons for this inequity [27]. Addressing this gap is vital, and strategies should not only enhance service delivery but also tackle educational and information access disparities to ensure equitable antenatal care for all economic groups.

## Strengths & limitations

Our study has several strengths. First, to our knowledge, this is the first study that examined the contents of ANC services from a nationally representative and large sample in Afghanistan; therefore, it will serve as a benchmark for further studies. Second, the use of data from a nationally representative survey with a larger sample may improve the external validity of this study, allowing the generalizability of the findings at the national level. Third, this study employed a questionnaire and study design similar to those employed in the demographic health surveys, and this may enable researchers to compare our findings with other LMICs.

There are several limitations in this study. First, recall bias may have occurred as women reported events and services from several months ago. They may not have recalled all the services they received. This may have resulted in underreporting of the contents of ANC services. Second, the women may have misclassified some of the health facilities from where or from whom they received the ANC services. Third, our study identified a significant association between knowledge of complicated pregnancy and the number of ANC services received, which is subject to measurement bias. It is possible that ANC services might have contributed to the knowledge of danger signs associated with complicated pregnancy. Fourth, our evaluation of predictors for the contents of antenatal care services received by a pregnant was restricted to the information documented in AHS dataset. Other important factors of relevance for the receipt of antenatal care contents such as distance to the health facility, pregnancy intention, and health expenditures were not assessed in the present study. Therefore, future studies should consider these potential confounders in their analyses. Fifth, the data analyzed in this study was collected in 2018. Therefore, the findings may not reflect current antenatal care practices, as the COVID-19 pandemic and the collapse of the internationally assisted Afghan government have greatly affected the delivery of healthcare services in Afghanistan [68, 69]. Sixth, there were only 27 nurses in our study, and this may have increased the chance of uncertainty in the results when we compared the delivery of 1–4 versus 5–8 services to pregnant women by nurses versus doctors, as well as by nurses versus midwives. Seventh, the study is limited by the AHS 2018’s scope, which did not encompass the entire spectrum of ANC services as recommended by the WHO in 2016. Consequently, several critical components of ANC necessary for more advanced stages of pregnancy, such as estimation of gestational age, assessment for multiple pregnancies, fetal positioning, determination of fetal heart rate, and consultations for birth preparedness or family planning, were not included in the services evaluated by this study. This exclusion might have impacted the comprehensiveness of our findings, particularly regarding care in the later trimesters. Finally, the associations observed between the contents of antenatal care services and its predictors in a cross-sectional study design does not account for causal inference.

## Conclusion

This study aimed to assess the contents of ANC services and identify predictors of utilization of services by pregnant women during ANC visits from health facilities in Afghanistan. We found that the contents of antenatal care services were underutilized and possibly not valued by the beneficiaries. We also found that women who could read and write, whose husbands could read and write, being primipara, more frequently watching TV, and having more knowledge of pregnancy complications were factors that positively influenced 5–8 antenatal care services utilization. On the other hand, women who received antenatal care services from a nurse and in a private clinic were less likely to receive 5–8 services of antenatal care.

Our findings on the type and number of ANC contents received by pregnant women raise causes for concerns and actions. Therefore, the Ministry of Public Health in Afghanistan, UN agencies, policymakers, and health professionals should give special attention to the contents of ANC services and intervene by not only focusing on healthcare provision but also by targeting uneducated pregnant women, uneducated husbands, and being nullipara pregnant women for health promotion through public campaigns, health education programs, and various media channels. In addition, tackling the underlying social determinants, including access to education, and gender equality are crucial. By addressing these multifaceted challenges, Afghanistan can move toward meeting national standards for the utilization of ANC contents and safeguarding the health and well-being of its pregnant women and their infants.

### Electronic supplementary material

Below is the link to the electronic supplementary material.

**Supplementary Material 1**: Comparison of the baseline characteristics of the 186 women who did not report a specific service with women who received services

## Data Availability

The data used to support the findings of this study can be requested from the Ministry of Public Health of Afghanistan (contact person: Dr. Saeedzai atasayedzai@gmail.com).
