# Supplementary material for: Contents of antenatal care services in Afghanistan: findings from the national health survey 2018
Source: BMC Public Health. 2023 Dec 11;23:2469. doi: 10.1186/s12889-023-17411-y (PMC10712111; doi:10.1186/s12889-023-17411-y)
Supplement: Supplementary file 1 — Supplementary Material 1: Comparison of the baseline characteristics of the 186 women who did not report a specific service with women who received services [file 12889_2023_17411_MOESM1_ESM.docx]

| Supplementary Table. Comparison of baseline characteristics of women with 0 service, 1-4 services, and 5-8 services | | | | | |
| --- | --- | --- | --- | --- | --- |
|  |  | Women with  0 service n=186(2.7%) | Women with  1-4 services n=4,544(66.7%) | Women with  5-8 services n=2,083(30.6%) | Total women  n=6,813(100%) |
| Age | |  |  |  |  |
|  | 14-29 years | 130(69.9%) | 2861(63.0%) | 1309(62.8%) | 4300(63.1%) |
|  | 30-39 years | 44(23.7%) | 1375(30.3%) | 642(30.8%) | 2061(30.3%) |
|  | 40-49 years | 12(6.5%) | 308(6.8%) | 132(6.3%) | 452(6.6%) |
| **Woman reads and writes | | 26(14.0%) | 861(19.0%) | 578(27.8%) | 1465(21.5%) |
| **Husband reads and writes | | 69(37.1%) | 1758(38.7%) | 948(45.5%) | 2775(40.7%) |
| Parity | |  |  |  |  |
|  | Nullipara (no childbirth previously) | 11(5.9%) | 200(4.4%) | 71(3.4%) | 282(4.1%) |
|  | Primipara (1 childbirth previously) | 24(12.9%) | 565(12.4%) | 293(14.1%) | 882(13.0%) |
|  | Multipara (≥2 childbirths previously) | 151(81.2%) | 3779(83.2%) | 1719(82.5%) | 5649(82.9%) |
| **Health provider | |  |  |  |  |
|  | Doctor | 76(40.9%) | 1793(39.5%) | 601(28.9%) | 2470(36.3%) |
|  | Midwife | 107(57.5%) | 2727(60.0%) | 1479(71.0%) | 4313(63.3%) |
|  | Nurse | 3(1.6%) | 24(0.5%) | 3(0.1%) | 30(0.4%) |
| **Health facility | |  |  |  |  |
|  | MoPH clinic | 60(32.3%) | 1640(36.1%) | 910(43.7%) | 2610(38.3%) |
|  | MoPH hospital | 25(13.4%) | 662(14.6%) | 430(20.6%) | 1117(16.4%) |
|  | Private clinic | 101(54.3%) | 2242(49.3%) | 743(35.7%) | 3086(45.3%) |
| **Number of danger signs the woman knew | | |  |  |  |
|  | None (symptom) | 120(64.5%) | 2066(45.5%) | 197(9.5%) | 2383(35.0%) |
|  | 1 symptom | 31(16.7%) | 1190(26.2%) | 640(30.7%) | 1861(27.3%) |
|  | 2 symptoms | 28(15.1%) | 965(21.2%) | 823(39.5%) | 1816(26.7%) |
|  | ≥ 3 symptoms | 7(3.8%) | 323(7.1%) | 423(20.3%) | 753(11.1%) |
| *Socioeconomic status | |  |  |  |  |
|  | Lowest (quintile) | 24(12.9%) | 621(13.7%) | 278(13.4%) | 923(13.6%) |
|  | Low | 29(15.6%) | 830(18.3%) | 338(16.2%) | 1197(17.6%) |
|  | Middle | 56(30.1%) | 934(20.6%) | 439(21.1%) | 1429(21.0%) |
|  | High | 41(22.0%) | 1031(22.7%) | 507(24.3%) | 1579(23.2%) |
|  | Highest (quintile) | 36(19.4%) | 1128(24.8%) | 521(25.0%) | 1685(24.7%) |
| Residence | |  |  |  |  |
|  | Urban | 47(25.3%) | 1191(26.2%) | 524(25.2%) | 1762(25.9%) |
|  | Rural | 139(74.7%) | 3353(73.8%) | 1559(74.8%) | 5051(74.1%) |
| Access to the media (almost daily) | |  |  |  |  |
|  | **Internet access | 4(2.2%) | 133(2.9%) | 93(4.3%) | 230(3.4%) |
|  | *Radio access | 40(21.5%) | 1187(26.1%) | 594(28.5%) | 1821(26.7%) |
|  | **TV access | 50(26.9%) | 1697(37.4%) | 921(44.2%) | 2668(39.2%) |
|  | *p-value < 0.05, **p-value < 0.01 | | | | |
